# Expression of Concern: Paladugu et al. Liraglutide Has Anti-Inflammatory and Anti-Amyloid Properties in Streptozotocin-Induced and 5xFAD Mouse Models of Alzheimer’s Disease. *Int. J. Mol. Sci.* 2021, *22*, 860

**DOI:** 10.3390/ijms26104467

**Published:** 2025-05-08

**Authors:** Internation Journal of Molecular Sciences Editorial Office

**Affiliations:** MDPI, St. Alban-Anlage 66, 4052 Basel, Switzerland; ijms@mdpi.com

With this notice, the *Internation Journal of Molecular Sciences* Editorial Office states their awareness of the concerns regarding the integrity of a number of the figures published within this publication [1]. Following discussion with the authors and a representative from the Central Michigan University, the Editorial Board has decided to allow the authors to confirm the validity of the overall findings by performing re-experimentation on certain elements of this study. As such, this Expression of Concern will stay in place until this process has been completed, and the Editorial Office will provide an update on this situation and announce any post-publication modifications deemed to be necessary, as per MDPI’s Update to Published Papers policy.
